# Considerations for Experimental Animal Models of Concussion, Traumatic Brain Injury, and Chronic Traumatic Encephalopathy—These Matters Matter

**DOI:** 10.3389/fneur.2017.00240

**Published:** 2017-06-01

**Authors:** Mark W. Wojnarowicz, Andrew M. Fisher, Olga Minaeva, Lee E. Goldstein

**Affiliations:** ^1^Molecular Aging and Development Laboratory, Boston University School of Medicine, Boston, MA, United States; ^2^Boston University College of Engineering, Boston, MA, United States; ^3^CTE Program, Boston University Alzheimer’s Disease Center, Boston, MA, United States

**Keywords:** traumatic brain injury, chronic traumatic encephalopathy, concussion, animal models, neurotrauma

## Abstract

Animal models of concussion, traumatic brain injury (TBI), and chronic traumatic encephalopathy (CTE) are widely available and routinely deployed in laboratories around the world. Effective animal modeling requires careful consideration of four basic principles. First, animal model use must be guided by clarity of definitions regarding the human disease or condition being modeled. Concussion, TBI, and CTE represent distinct clinical entities that require clear differentiation: concussion is a *neurological syndrome*, TBI is a *neurological event*, and CTE is a *neurological disease*. While these conditions are all associated with head injury, the pathophysiology, clinical course, and medical management of each are distinct. Investigators who use animal models of these conditions must take into account these clinical distinctions to avoid misinterpretation of results and *category mistakes*. Second, model selection must be grounded by clarity of purpose with respect to experimental questions and frame of reference of the investigation. Distinguishing *injury context* (“inputs”) from *injury consequences* (“outputs”) may be helpful during animal model selection, experimental design and execution, and interpretation of results. Vigilance is required to rout out, or rigorously control for, model artifacts with potential to interfere with primary endpoints. The widespread use of anesthetics in many animal models illustrates the many ways that model artifacts can confound preclinical results. Third, concordance between key features of the animal model and the human disease or condition being modeled is required to confirm model biofidelity. Fourth, experimental results observed in animals must be confirmed in human subjects for model validation. Adherence to these principles serves as a bulwark against flawed interpretation of results, study replication failure, and confusion in the field. Implementing these principles will advance basic science discovery and accelerate clinical translation to benefit people affected by concussion, TBI, and CTE.

## Introduction to Animal Modeling of Human Disease

### Why Do We Need Animal Models of Human Disease?

Animal models of human diseases represent essential tools in the biomedical research armamentarium (1–3). Animal models provide time-tested tools to establish causal mechanisms of disease, facilitate hypothesis testing, enable systematic exploration of pathophysiology, and conduct rigorously controlled “proof of concept” experiments to evaluate new diagnostics and therapeutics that would be impractical or unethical in humans. Animal models are deployed for many purposes, including (i) identification of disease substrates, pathways, and mechanisms; (ii) elucidation of genotype–phenotype relationships; (iii) validation of diagnostic biomarkers and therapeutic targets; and (iv) development and testing of new treatments prior to launching costly clinical trials. Given the availability of model organisms suitable for genetic manipulation (mice, fish, flies, nematodes), laboratory animals also provide unparalleled opportunities for dissecting genotypic contributions to many different human diseases (4–8) and identifying mechanistic pathways that may be targetable for personalized therapeutic intervention (9, 10). *A compelling argument can be made that animal models provide the single most effective means for translation of basic science discovery into clinical advances that benefit human patients* (3, 11).

On the other hand, animal models are constrained by the obvious fact that model organisms are not human. This argument is often presented as an inherent limitation of experimental use of animal models of human diseases. While this position has undeniable face validity, evolutionary considerations provide a compelling counterargument. Consider the laboratory mouse (*mus musculus*). While human and mouse lineages diverged from a common ancestor ~75 million years ago, virtually all human coding genes have homologous counterparts in the mouse genome (12). In addition, ~96% of coding genes localize to highly conserved syntenic regions of the genome in both species. These surprising observations mean that not only do mice and humans share substantial overlapping homology in coding genes but also that long blocks of homologous genes appear in the same order (synteny) in both species. Gene structure, including the number and coding length of exons and non-coding sequences, is also highly conserved. Moreover, gene homologs (and also gene orthologs that diverged from a common ancestor) code for proteins with identical or closely related physiological functions in both species. Mice and humans also share similar anatomical organization of cells, tissues, and organs, including the brain. The two species also share substrates and mechanisms underpinning reproduction, embryogenesis, organogenesis, maturation, homeostasis, and senescence—even neurophysiology and behavior. In addition to genotypic and anatomical similarities with humans, mice (and other model organisms) afford many practical advantages, including experimental malleability, ease of breeding and housing, and straightforward genetic manipulation (13). These considerations make the mouse an unparalleled model organism to investigate mechanisms of human diseases and bridge the chasm between bench science and clinical medicine.

Despite these merits, preclinical research that utilizes animal models has come under intense scrutiny (14–18). Criticisms include lower than expected study replication rates, interpretive issues regarding generalizability of results to humans, and failure of massive federal investment to deliver clinical returns. These concerns have captured the attention not only of the biomedical research community but also decision makers in the pharmaceutical industry and federal funding agencies, including the National Institutes of Health (NIH) (19). Spirited debate has ensued among stakeholder constituencies, including laboratory scientists, clinical investigators, patient advocates, scientific organizations, and animal rights activists. Collectively, these concerns have triggered calls to recalibrate biomedical research by focusing on clinical investigation.

### Complementary Nature of Preclinical and Clinical Research

Such suggestions, explicitly stated or implied, are misplaced and confuse the different objectives of preclinical and clinical research. We argue here [as we have elsewhere (3)] that preclinical and clinical research are inextricably linked, mutually reinforcing, and fundamentally complementary. However, the aims of clinical investigation are categorically different from those that drive preclinical research. We described objectives of preclinical research. By contrast, clinical studies, particularly when descriptive or retrospective, are designed to categorize human disease (clinical nosology), identify disease phenotypes (clinical description), and correlate disease features with clinical pathology, medical history, genetics, environmental exposure, and outcome measurements (clinical correlation). Prospective clinical studies are often designed to comparatively evaluate diagnostics, treatments, prophylactic measures, rehabilitative strategies, or clinical interventions with regard to clinical safety, tolerability, or efficacy. Such studies are typically conducted in a defined set of patients with a given disease, risk factor, or medical history. Results obtained by clinical investigation are essential to advance medical knowledge and promote safe and effective medical care. However, clinical investigation is often constrained by ethical barriers, cohort heterogeneity, individual variation, control group inadequacies, selection and stratification biases, sample size, study reproducibility and generalizability, and perhaps most importantly, inherent limitations of clinical correlation to establish mechanistic causality (3, 20). These factors, alone or in combination, can lead to clinical results that are incremental, incomplete, or inconclusive.

*These considerations underscore the need to integrate mechanistically-directed preclinical research that incorporates animal modeling with complementary clinical investigation involving human subjects* (3). The case we make here is simple. For animal models of concussion, TBI, and CTE to be clinically informative and translationally useful, these models must be grounded by: (i) clarity of definitions with respect to the human disease or condition being modeled (nosology), (ii) clarity of purpose with respect to how the animal model will be used (utility), (iii) concordance between features of a given animal model and the human disease or condition being modeled (biofidelity), and (iv) confirmation of experimental results obtained in laboratory animal with clinical findings in human subjects (validity).

## Clarity of Definitions

Establishing clear definitions of the clinical condition being modeled represents a prerequisite for any useful animal model. Indeed, *clarity of clinical definitions is arguably the single most important consideration for animal modeling of any human disease or condition*. Without clear clinical definitions, animal models of human conditions will founder with respect to biofidelity (“what is the animal model modeling”) and validity (“how well do experimental results in animals map to clinical findings in humans”). Finally, clear clinical definitions serve an important secondary function, namely, to prevent category mistakes. A category mistake (or fallacy) is a logical error in which entities representing a specific set of attributes is confused or conflated with another entity representing related but non-overlapping attributes. The philosopher Gilbert Ryle famously described a visitor to Oxford who after touring the colleges, library, and landmarks asked, “But where is the University?” (21). The visitor’s logical mistake derives from the erroneous assumption that the “University” is represented by a categorical set of physical structures (e.g., Magdalen Tower) rather than organizational relationships (e.g., University of Oxford). Similarly, the three clinical entities considered here (concussion, TBI, and CTE) are each associated with head injury but represent distinct clinical categories that require conceptual (nosological) differentiation: concussion is a *neurological syndrome*, TBI is a *neurological event*, and CTE is a *neurological disease*. Confusion regarding such distinctions leads to category mistakes, flawed interpretation of results, confusion in the field, and scientific miscommunication (3, 20). Here we argue that the formulation “concussion is a TBI” represents such an error (22). As noted by Kuhn, conceptual incompatibility fosters scientific incommensurability and undermines the routine practice of normal science (23).

Clear and accurate definitions are not simply academic niceties. Rather, the clarity of definitions principle serves as the foundation on which animal modeling and experimental science are built. Definitions, whether explicitly stated or implicitly assumed, have real consequences in the real world. These matters matter.

### Concussion

**Figure 1:**
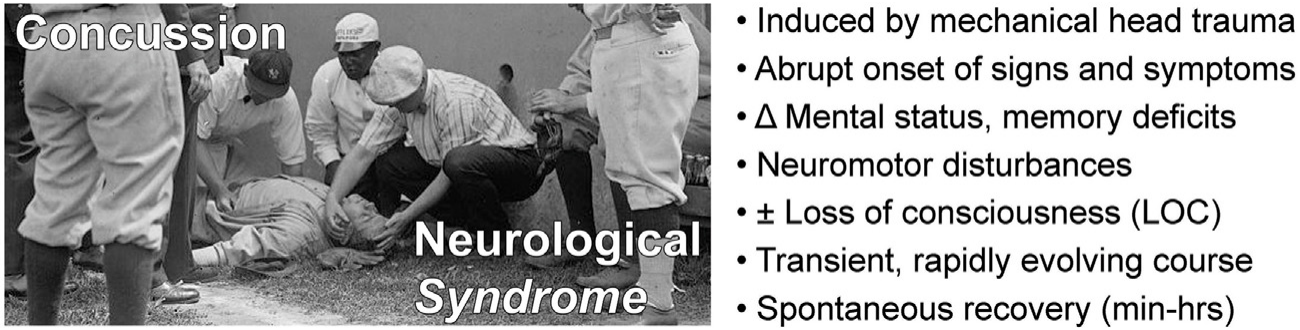
Concussion is a *neurological syndrome* defined by an inciting head injury that triggers abrupt onset of a clinically defined constellation of transient signs and symptoms that spontaneously resolves over a typical course of minutes to hours.

*Concussion is a neurological syndrome defined by abrupt onset of a constellation of transient neurological signs and symptoms triggered by head trauma* (Figure 1). Conceptualizing concussion as a “syndrome” (from Greek, sundromē, to run together) captures the concurrence of an incident head injury with onset of a set of signs (observed by others) and symptoms (reported by the subject) that define this condition. Classifying concussion as a neurological syndrome also provides a conceptual framework to distinguish this condition from other head injury-related conditions with which concussion is often confused (TBI, a *neurological event*; post-concussion syndrome, a *neurological sequela*; CTE, a *neurological disease*).

The first recorded description of concussion is attributed to the Persian physician Muhammad ibn Zakariya al-Razi (Rhazes, c. 853–929 CE), chief physician of Baghdad, who noted the abrupt onset and transient nature of the disorder, and additionally, appreciated that concussion can occur independently of penetrating injury, skull fracture, or gross brain pathology (24, 25). Centuries later, Ambroise Paré (1510–1590), a French military surgeon, increased medical awareness and diagnostic recognition of concussion in Europe. The origin and popularization of the word “concussion” (from Latin *concussus*, to strike together; or *concutere*, to shake violently) date to this period (26). Concussion is synonymous with *commotio cerebri*, an archaic term that is occasionally used to describe “commotion” (violent agitation) of the brain resulting from head injury (27, 28). An important early observation regarding the pathophysiology of concussion is attributed to Alexis Littré (1654–1726), a French physician and anatomist, who conducted a celebrated postmortem examination of a concussed criminal who repetitively banged his head against his prison cell wall in anticipation of execution. To the surprise of his medical colleagues, Littré’s examination did not reveal evidence of brain injury, thus confirming his belief that concussion reflects a transient disturbance of neurological function rather than structural brain injury (29). The classical formulation of concussion as a clinical syndrome was penned in 1787 by Benjamin Bell (1749–1806), a renowned neurosurgeon at the Edinburgh Infirmary. Bell described the condition as an “…*affection of the head attended with stupefaction, when it appears as the immediate consequence of external violence, and when no mark or injury is discovered, is in general supposed to proceed from commotion or concussion of the brain, by which is meant such a derangement of this organ as obstructs its natural and usual functions, without producing such obvious effects on it as to render it capable of having its real nature ascertained by dissection*.” More than two centuries have passed, yet nothing written before or since captures the condition so clearly.

Modern definitions of concussion include the following components: (i) temporal association with an antecedent head trauma; (ii) rapid onset of neuropsychiatric, cognitive, and neuromotor deficits; (iii) absence of demonstrable structural brain injury; and (iv) transient signs and symptoms that spontaneously resolve, typically over minutes to hours (30–33). The 4th International Conference on Concussion in Sport (November 2012) held in Zurich, Switzerland, defined concussion as a pathophysiological process affecting the brain that is induced by biomechanical forces and results in clinical symptoms (e.g., headache), neuromotor signs (e.g., unsteadiness), impaired brain function (e.g., confusion, amnesia, executive dysfunction), or abnormal behavior (e.g., personality or mood disturbances). Significantly, and in accordance with other recent consensus definitions, the Zurich formulation does not require loss of consciousness (LOC) as a defining feature of concussion (32, 34). Position statements on the definition of concussion have been promulgated by other organizations, including the American Medical Society for Sports Medicine (AMSSM) (31) and American Academy of Neurology (AAN) (30). The most commonly used standardized test to identify a concussion is the Sport Concussion Assessment Tool 3 (SCAT3) (35). SCAT3 uses the Glasgow Coma Scale (GCS) to assess consciousness, Maddocks score to assess orientation (person, place, and time), and other tests to evaluate balance, orientation, memory, concentration, and physical symptoms such as headache. The SCAT3 evaluation is generally consistent with other definitions of concussion (36). A detailed discussion of the history and pathophysiology of concussion can be found in comprehensive reviews (25, 26). A summary of key clinical features of the concussion syndrome is provided in Figure 1.

### Traumatic Brain Injury (TBI)

**Figure 2:**
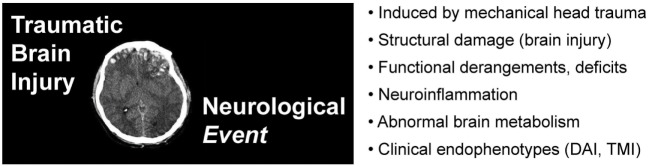
Traumatic brain injury (TBI) is a *neurological event* defined by an inciting head trauma that results in structural brain damage and neurological dysfunction. TBI implies structural brain pathology (neurological or brain imaging lesions) and/or alterations in brain function (neurological impairment). Image adapted from Ref. (37).

Traumatic brain injury is a neurological event marked by structural, cellular, molecular pathology, and/or functional disturbances in the central nervous system triggered by head trauma [Figure 2, image adapted from Ref. (37)]. For the purpose of this brief overview, we will focus on closed-head injuries that fall within the mild end of the TBI severity spectrum. Mild forms of TBI represent the overwhelming majority of such injuries in the US and worldwide (38–40). By definition, these injuries are not associated with structural brain lesions, and in uncomplicated cases, do not typically warrant brain imaging examination (e.g., computed tomography, magnetic resonance imaging) or require inpatient hospitalization (41–43). Diagnostic assessment is often based on a single post-injury evaluation that may not adequately rule out rare cases of an evolving neurological event or potentially life-threatening sequelae [e.g., intracranial hemorrhage, second impact syndrome (SIS)] (44). As a practical matter, conceptualizing TBI as a dynamic neurological process focuses clinical attention on evaluation of a potentially fluctuating course rather than diagnosis of a static condition.

By clinical convention, mild TBI is diagnosed in patients who have sustained a closed-head injury that results in a GCS score of 13–15 indicating minimal or no change in mental status (44). According to an influential American College of Rehabilitation Medicine (ACRM, 1993) position statement (45), mild TBI is defined as an alteration of brain function caused by external forces that results in one or more of the following clinical features: (i) change in mental status (confusion, disorientation, or slowed thinking), (ii) LOC lasting 0–30 min, (ii) post-traumatic amnesia (PTA) lasting less than 24 h; (iii) focal neurologic deficits that may or may not be transient, and (iv) GCS score of 13–15 at 30 min post-injury. The 1993 ACRM position statement was the first consensus definition of mild TBI that does not require LOC for diagnosis and also the first to recognize PTA as an independent diagnostic feature (43, 46). The World Health Organization (WHO, 2004) (47, 48) definition of mild TBI generally follows the 1993 ACRM criteria but does not include alterations in mental status for diagnosis. The US Centers for Disease Control and Prevention (CDC, 2003) (49) defines mild TBI as an insult to the head due to blunt contact or acceleration-deceleration of the head that results in one or more of the following clinical features: (i) transient confusion, disorientation, or impaired consciousness; (ii) amnesia or memory dysfunction around the time of injury; (iii) neurological or neuropsychological dysfunction (in adults: seizure, headache, dizziness, irritability, fatigue, or poor concentration; in infants and young children: irritability, lethargy, or vomiting);^1^ and (iv) LOC ≤30 min. Note that the 1993 ACRM and 2004 WHO definitions of mild TBI include GCS and PTA criteria, whereas the CDC does not require either component. Moreover, none of these consensus definitions require LOC. The US Department of Veteran Affairs and Department of Defense (VA-DoD, 2009) (50) issued a Clinical Practice Guideline that included a definition of mild TBI that generally follows the 1993 ACRM criteria with an added neuroimaging qualification: (i) LOC <30 min, (ii) alteration of consciousness <24 h, (iii) post-traumatic amnesia <1 day, (iv) initial GCS score between 13 and 15, and (v) normal structural brain imaging. All of these consensus definitions overlap significantly with one another and with more recent consensus formulations [National Academy of Neuropsychology, 2009 (51); International and Interagency Initiative Toward Common Data Elements for Research on Traumatic Brain Injury and Psychological Health, 2010 (41)].

Importantly, all of these definitions are consistent with classifying *TBI as a neurological event*. An influential recent position statement defined TBI “*as an alteration in brain function, or other evidence of brain pathology, caused by an external force*” (41). In this definition, as in others, the functional alteration or structural pathology in the brain is explicitly linked to an inciting insult exerted by an external force. This causal connection defines the neurological event. From this perspective, TBI is analogous to other common diagnostic formulations that connote sudden onset of tissue pathology, including “cerebrovascular accident” [CVA, “stroke” (52, 53)] and “acute myocardial infarction” [AMI, “heart attack” (54)] Each of these conditions is defined by a specific set of clinical signs, symptoms, and test results that follow a characteristic temporal course *without reference to the underlying etiology or mediating pathophysiology*. Stated simply, diagnosis of these conditions (as with TBI) is independent of etiology precisely because they are medical events, not diseases *per se*.

These considerations have practical implications for clinical care. While workups for CVA and AMI are based on established clinical definitions and validated medical protocols accepted around the world, the situation for TBI remains challenging and highly variable across geographic areas, institutions, and even between providers. Variation in clinical practice is especially notable at the mild end of the TBI spectrum. A number of factors contribute to this situation (41). First, diagnostic evaluation of TBI frequently relies on incomplete or unreliable information from the patient, family members, or witnesses collected during a single examination. Second, clinical evaluation may be clouded by direct effects of the injury, neuropsychiatric comorbidities, medication effects, recent substance use, psychogenic and/or psychosocial stress, practical concerns (e.g., return-to-play decision, medicolegal liability), and secondary gain that, alone or in combination, may distort accurate reporting by patients and informants. Finally, the diagnosis is currently conferred without reference to validated biomarkers that are sensitive and specific for TBI. For all of these reasons, diagnosis and management of TBI, especially when mild, remains clinically challenging and medically controversial.

#### Diffuse Axonal Injury (DAI)

Diffuse axonal injury is a TBI endophenotype that is characterized by structural and/or functional abnormalities that affect the integrity of axons and nerve fibers in the brain (55). DAI is induced by intraparenchymal shearing forces that mechanically disrupt axon structure (structural axotomy) and/or axon function (functional axotomy). Injury to specific axons and nerve fiber bundles may include chronic and progressive pathologies, including retraction bulbs (56, 57). DAI also interferes with axon physiology, axoplasmic transport, microtubule organization, and molecular interactions of axonal proteins, including the microtubule-associated protein tau (MAPT, tau). DAI can be highly focal or diffusely generalized depending on trauma severity, injury biomechanics, local neuroanatomy, and host factors. The pioneering studies of Sabina Strich were amongst the first to rigorously perform clinicopathological correlation analysis focusing on DAI (58, 59). Neuropathological examinations in these cases showed evidence of white matter retraction bulbs and axonal spheroids revealed by silver staining. DAI is often accompanied by disruption of axoplasmic transport (60, 61). Evidence of DAI can be visualized by immunohistological detection of abnormal accumulations of the amyloid precursor protein (APP) (62, 63), a fast transport cargo present in most axons in the brain. DAI is also detectable *in vivo* by diffusion tensor imaging (64–67).

#### Traumatic Microvascular Injury (TMI)

Traumatic microvascular injury is another TBI endophenotype that is characterized by damage to small blood vessels in the brain (68, 69). TMI has received far less attention than DAI in the medical literature and as a consequence is underappreciated as a neurological consequence of neurotrauma. Like nerve fibers, small blood vessels in the brain are vulnerable to mechanical injury resulting from intraparenchymal shearing forces. Structural damage to the brain microvasculature can result in blood flow occlusion (stasis), blood–brain barrier (BBB) disruption, reactive neuroinflammation, and frank hemorrhage (microbleeds). Functional sequelae associated with TMI include vasogenic edema, plasma protein extravasation, altered cerebrovascular reactivity, abnormal neurovascular coupling, spreading depolarization, post-traumatic seizure, and neurocognitive impairment. As with DAI, TMI can be highly focal or diffusely generalized depending on trauma severity, injury biomechanics, local neuroanatomy, and host factors. In rare instances, a TBI sustained during recovery from a prior insult can trigger SIS, a catastrophic neurovascular condition associated with diffuse intracerebral hemorrhages, cerebrovascular autoregulatory failure, global brain swelling, herniation, vascular collapse, and death (70, 71).

While SIS is an exceedingly rare, emerging data indicate that TMI may be a common TBI endophenotype, even in cases of mild injury (68, 69). Recent studies have shown that contact sport athletes with mild TBI demonstrate elevated blood levels of CNS-derived proteins, including S100 calcium-binding protein B (S100B), neuron-specific enolase (NSE), neurofilament light chain protein (NFL), and tau protein (including various phosphorylated tau proteoforms and cleavage products) (72–74). Investigators in Israel recently used dynamic contrast-enhanced magnetic resonance imaging (DCE-MRI) to detect and map TBI-related cerebrovascular permeability defects in amateur American football players after a session of play (75). Taken together, these findings indicate that even mild TBI may be associated not only with structural damage involving brain cells and their processes but also with microvascular injury and BBB breakdown that results in release of brain cell products into the peripheral circulation. Development of new ultrasensitive biomarker assays designed to detect elevated blood levels of brain proteins, lipids, and even subcellular products (e.g., exosomes) affords a promising strategy for TBI diagnosis, prognosis, and monitoring (74, 76–79).

#### Other TBI Features

Neuroinflammation is a cardinal pathophysiological feature of TBI (80–82). Perivascular microgliosis and diffuse astrocytosis have been reported in human brain within hours of TBI (83). Post-traumatic neuroinflammation may persist chronically (84, 85). Microglia are known to migrate to sites of brain injury where these cells undergo phenotypic transformation and activation (86–89). Activated microglia secrete a variety of both pro- and anti-inflammatory cytokines (90–93). TBI patients also express autoreactive antibodies that may prove to be useful biomarkers of brain injury (94, 95). In addition to activating neuroinflammatory responses, TBI also disrupts normal homeostatic control of cerebral metabolism. TBI triggers a pathological neurometabolic cascade that impairs brain function and increases cerebral vulnerability to subsequent brain insults (96–98). Acute neurotrauma stimulates local release of glutamate, an excitatory neurotransmitter that depolarizes neurons and increases metabolic demand of membrane pumps needed to restore physiological ion concentrations (99, 100). The resulting increased brain energy demand disrupts oxidative metabolism, thereby increasing lactate production (via anaerobic glycolysis), acidosis, and brain edema (96–98).

### Chronic Traumatic Encephalopathy (CTE)

**Figure 3:**
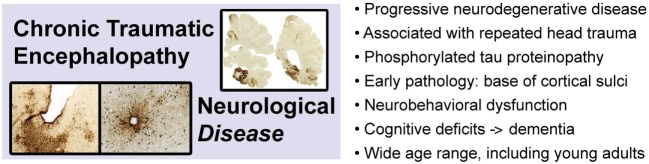
Chronic traumatic encephalopathy is a slowly progressive *neurodegenerative disease* associated with repetitive head injury exposure and defined by a distinctive pattern of phosphorylated tau protein pathology in the brain. Images adapted from Ref. (106).

Chronic traumatic encephalopathy is a slowly progressive neurodegenerative disease associated with repetitive neurotrauma exposure and defined by a unique pattern of phosphorylated tau protein neuropathology (Figure 3) (3, 101–106). CTE is invariably associated with repetitive head injury, most commonly sports-related head injuries or military-related blast exposure. The disease manifests clinically as the traumatic encephalopathy syndrome (TES), a slowly progressive condition characterized by mood and affect disturbances, cognitive impairment, and ultimately, frank dementia (107–109). The risk of developing CTE (and TES) increases with duration, age of first neurotrauma exposure, and advancing age, but significantly, does not correlate with number of concussions (110, 111). Indeed, CTE has been diagnosed in subjects with histories of repetitive head trauma but without frank concussion (111). These observations suggest that repetitive neurotrauma (“hits”), rather than concussion *per se*, is the causal trigger that initiates CTE pathophysiology. At present, definitive diagnosis of CTE can only be made by postmortem neuropathological examination. The pathognomonic lesion (defining hallmark) of CTE is the presence of perivascular accumulations of abnormally phosphorylated tau protein at the base (“depths”) of cortical sulci (105). This lesion and the uniquely defining pattern of neuropathology in CTE have been identified in individuals across a broad age range, including teenagers and young adults (112, 113) and is not observed in other tauopathies (e.g., Alzheimer’s disease, progressive supranuclear palsy, corticobasal degeneration, frontotemporal lobar degeneration, and variant disorders) or other neurodegenerative diseases (104, 105, 114). Tau protein is normally expressed in neurons, binds to and stabilizes microtubules, and promotes tubulin assembly (115, 116). Aberrantly phosphorylated tau protein interferes with these functions and is prone to pathogenic protein aggregation and gain-of-function neurotoxicity. While other neuropathological features may be present (e.g., TDP-43 immunoreactive neurons, dilatation of the third ventricle, septum cavum pellucidum, mammillary body atrophy, and signs of prior brain contusion), the diagnosis of CTE requires evidence of deep sulcal perivascular tau pathology (105). The earliest stage of CTE neuropathology manifests as isolated cortical lesions, typically in frontal cortex, that may be clinically asymptomatic or accompanied by minor neuropsychiatric impairment. The distinctive pathology and localization of early lesions likely reflect the injurious effects of mechanical stress concentration at the base of cortical sulci, around small blood vessels, and along gray–white matter interfaces (112, 117, 118). Focal injury induces local tau protein phosphorylation, aggregation, and miscompartmentalization that lead to neuronal dysfunction and progressive neurodegeneration (119–121). Clinical progression follows a characteristic pattern of regional spread of the underlying tau proteinopathy. Hyperphosphorylated tau protein is intrinsically neurotoxic and additionally, appears to spread throughout the brain. Various mechanisms for this spread have been proposed, including prion-like protein templating, trans-synaptic communication, exosomal secretion, glial cell processing, glymphatic dissemination, and other pathways (122, 123). The cellular pathobiology and molecular mechanisms underpinning CTE tau proteinopathy and spread are poorly understood and under intensive investigation. Animal modeling is a critical and irreplaceable component of this research effort.

## Clarity of Purpose: Animal Models of Concussion, TBI, and CTE

A number of recent reviews provide excellent overviews of TBI animal models (124–129). This review will focus on general issues pertinent to effective use of animal models. The issues discussed below are generally applicable to a wide range of animal models, including those designed for concussion, TBI, and CTE research. The major point here is that the validity and utility of any given animal model are not an intrinsic property of the model. The statement *“This animal model is valid, that one is not”* is meaningless without reference to the specific purpose and frame of reference of the model. *The validity and utility of a given animal model critically depend on purpose and frame of reference*. Maintaining clarity of model purpose, frame of reference, and limitations represent critical prerequisites for scientifically informative and clinically useful animal modeling.

### “Input” and “Output” Frames of Reference

**Figure 4:**
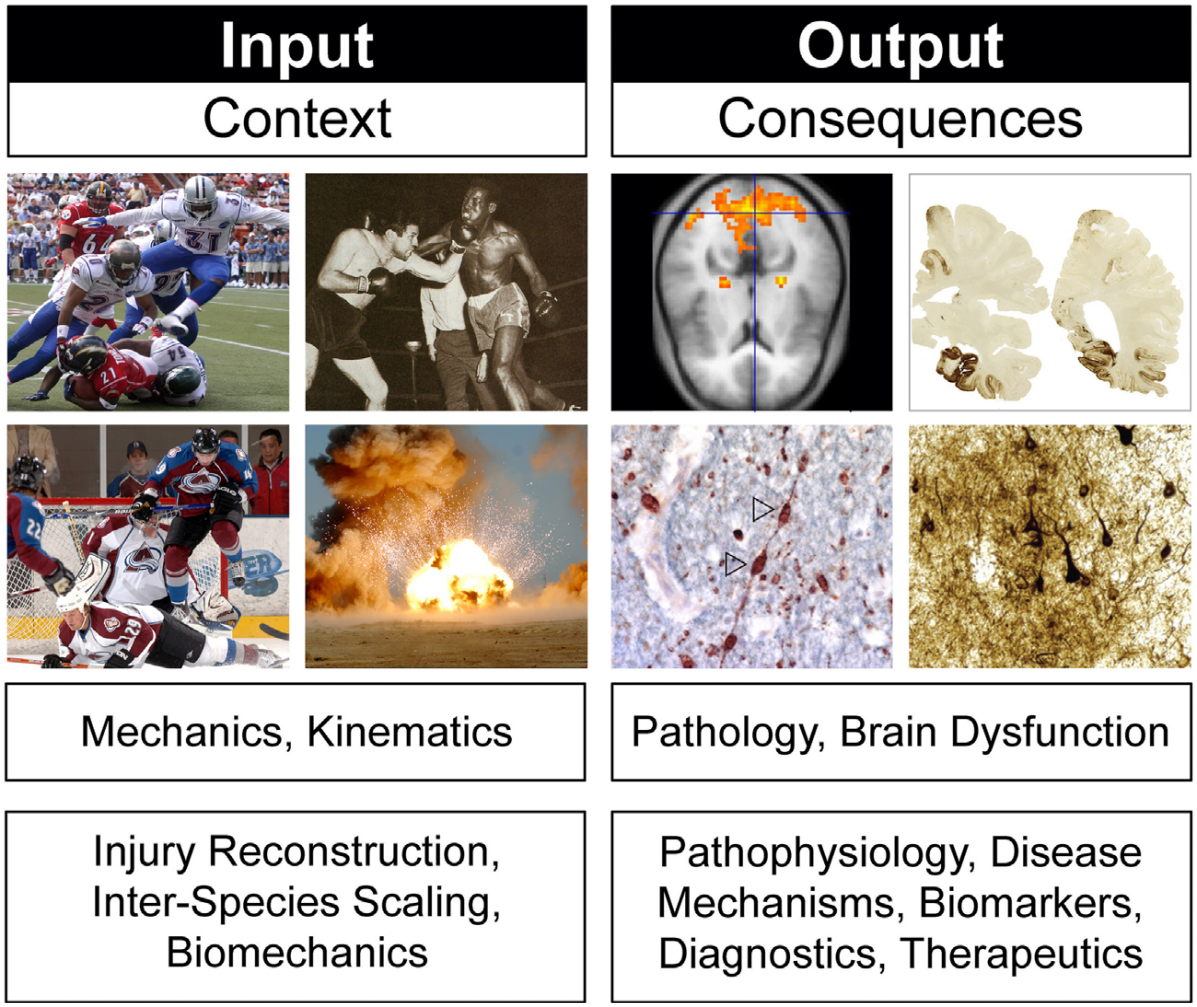
Frames of reference in animal models of concussion, traumatic brain injury, and chronic traumatic encephalopathy. Different frames of reference, illustrated here by head injury *context* (“input”) and head injury *consequences* (“output”), establish different criteria for evaluating model biofidelity, validity, and utility. Frames of reference and model validity, biofidelity, and utility are defined by reference to experimental purpose. See text for details. Brain pathology images adapted from Ref. (106).

Animal models of concussion, TBI, and CTE can be viewed through two overlapping but distinct frames of reference (Figure 4). The first focuses on *experimental conditions* (“input”) while the second considers *experimental consequences* (“output”). This distinction is often evident in the rationale for selecting a particular TBI animal model to address a particular experimental question. Justification may appeal to specific model features or parameters and (an often implicitly stated) frame of reference. For example, an animal model of closed-head impact injury might be biomechanically scaled to match reconstruction data from human head injuries (130). Similar strategies have been used to justify selection of particular blast neurotrauma models (131–133). Experimental utilization of such animal models incorporates a frame of reference that prioritizes *injury context* (“input”; Figure 4). Experimental design based on an “input” frame of reference is scientifically sound so long as the *purpose of the model* is to investigate biomechanical questions that require scaling to reach scientifically valid conclusions. However, an accurately scaled animal model that does not produce brain pathology similar to that in humans would be inappropriate for experiments that target TBI pathobiology. For this purpose, other animal models designed with a frame of reference that targets *injury consequences* (“output”; Figure 4) would be more informative. Justification of “output” models relies on concordance with brain pathology, neuroimaging, and behavioral deficits in humans (112, 120, 134). Stated differently, validity and utility of these models are based on the degree to which the model recapitulates clinical features and pathophysiological mechanisms of the human disease being modeled (3). *Experimental use of “output” models is scientifically appropriate when the purpose of the animal model targets brain pathology, disease mechanisms, neurobehavioral dysfunction, and diagnostic-therapeutic development* (3). Still other animal models draw from both perspectives and are constrained accordingly. The important point here is that *model validity critically depends on model purpose and frame of reference* (3, 135–138). This principle implies that model validity is not an intrinsic property of the model, but rather a dependent condition of purpose and frame of reference. Theoretical and practical implications of these considerations have been reviewed elsewhere (3).

### Model Artifacts: Anesthesia As a Case Example

Clarity of purpose extends beyond model selection. Vigilant attention is required throughout experiment design and execution to identify and rout out (or rigorously control) technical artifacts with potential to modulate or interfere with mechanisms, phenotypes, or endpoints under investigation. The widespread use of anesthesia in many animal models illustrates the ways in which a near-universal (and often overlooked) technical feature of many animal models can complicate experimental studies. Anesthesia artifacts represent an especially problematic area of concern for preclinical studies that incorporate animal models to investigate concussion, TBI, and CTE. A partial list of potential anesthesia-related artifacts underscores this point: (i) surgical-depth anesthesia (regardless of agent or route of administration) causes profound depression of the sensorium, level of arousal, muscle tone, reflex arc reactivity, and other vegetative neurological functions (139), thus complicating or precluding meaningful neurological assessment of concussion; (ii) commonly used anesthetics such as ketamine are pharmacologically active at glutamate receptors, thereby altering excitatory neurotransmission that underpins basic functions of the brain (140–142); (iii) many anesthetics also modulate neuroprotection and programmed cell death in the brain (143–145); (iv) commonly used anesthetics, such as xylazine, potently depress respiration, induce bradycardia and hypotension, and modulate neurochemical and neuroinflammatory responses after neurotrauma (even brief episodes of cerebral hypoxia trigger neuronal demise, i.e., pathoklisis, in vulnerable brain regions, including the hippocampus); (v) brief exposure to anesthesia modulates neuropathological outcomes in an agent-dependent manner following experimental neurotrauma in mice (146); and (vi) anesthesia potentiates tau protein phosphorylation in an agent-independent manner and this effect may persist long after anesthetic exposure (147–149). This list provides justification for revising animal model protocols to include components without anesthesia for critical experiments that may be compromised by anesthetic exposure.

## Concordance and Confirmation of Animal Models of Concussion, TBI, and CTE

Demonstrating concordance between specific features of a given animal model and the human disease or condition being modeled (i.e., biofidelity) goes hand in hand with confirmation of experimental results across different animal models with clinical findings in humans (i.e., validity). Combining these approaches is especially important when the experimental objective is focused on injury consequences (“output”). In this section, we briefly survey a number of commonly used animal models suitable for confirmatory studies, then close by summarizing the basic principles outlined in this review.

### Brief Survey of Concussion, TBI, and CTE Animal Models

#### Animal Models of Acute Concussion

Development of biofidelic animal models of concussion has been hampered by the near-universal protocol requirement for anesthesia during experimental neurotrauma [reviewed in Shaw (26)]. Time-dependent changes in reflex arcs or responses latencies are commonly used as neurological proxies for concussion. Alterations in a wide range of reflexes and neurovegetative responses—including corneal, papillary, and pinna reflexes; stretch and withdrawal responses; and alterations in blood pressure, pulse rate, and respiration—have also been used as proxy measures to assess experimental concussion in laboratory animals (26). Commonly used proxy metrics include latency of the righting reflex and time to spontaneous ambulation. Both indices are measured immediately after vapor anesthetic withdrawal and experimental neurotrauma (150–153). However, none of these proxy metrics accurately capture the constellation of neurological signs and symptoms that characterize acute concussion in humans (see “Concussion,” above; Figure 1). Prominent among the important missing domains are amnestic measures. Moreover, proxy metrics that use reflex arcs and response times are strongly modulated by antecedent exposure to systemic anesthetic. These include anesthetic concentrations in the blood and tissues at the moment of vapor anesthetic withdrawal, residual anesthetic in these compartments at the time of experimental head injury, and individual variation in anesthetic metabolism and pharmacodynamics. Additional complications may arise as a consequence of anesthetic retention in the setting of post-traumatic hypopnea, frank apnea, hypotension, bradycardia, and/or other cardiovascular and respiratory disturbances. These considerations seriously complicate interpretation of reflex latencies and motoric response times as proxy metrics for concussion. An obvious solution to this problem is to abandon anesthesia. Petraglia et al. (154) used a purpose-adapted neurotrauma mouse model and modified neurological severity scale to score neurological impairments in non-anesthetized mice after TBI. These investigators noted decrements in neuromotor performance (i.e., increased neurological injury severity) in mice following single and repeated TBI compared to uninjured controls. However, the study conducted evaluations starting 1 h post-injury. Based on the delayed timing of the initial evaluation and the specific domains tested, this study did not capture the course of acute concussion, but rather, of post-traumatic sequelae in the early (acute, sub-acute) time period post-injury. Unfortunately, few investigators have adopted this promising strategy. Development of new animal models of concussion without anesthesia represents an important unmet research need.

#### Animal Models of TBI

Fluid percussion injury (FPI) uses a reservoir of fluid to generate hydraulic pressure that impinges on a circumscribed area of craniotomy-exposed dura. Hydraulic pressure is generated within a fluid-filled piston driven by contact with a movable pendulum. The intensity of the pressure is controlled by adjusting the height of the released pendulum. FPI has been shown to induce diffuse brain injury and corresponding neurobehavioral deficits in a variety of laboratory animals, including mice, rats, cats, and pigs (56, 153, 155–157). FPI causes visible brain contusions with localized neuronal death in as little as 12 h post-injury. Microglial activation and astrocytosis (158, 159) are also notable following FPI. Cerebral hypoxia and death are complications of FPI, as this model injury induces pronounced post-injury apnea due to brainstem trauma (160, 161). FPI does not involve linear or rotational acceleration of the head, thus rendering this model most suitable for investigations of blunt-force trauma. Craniotomy is required for FPI and is known to induce localized neuroinflammation. As a consequence, craniotomy and sham FPI should be carried out in control animals. Both the craniotomy and FPI require anesthesia.

Controlled cortical impact (CCI) uses a pneumatic piston or electronic solenoid to drive an impactor through a craniotomy (intracranial CCI), or in an alternative configuration, onto the skull (extracranial CCI). Depth, duration, and force of impact on the dura or skull can be controlled and varied according to experimental need. CCI produces cortical tissue loss, blood–brain barrier disruption, neuroinflammation, axonopathy, and contusion (162–165). CCI also induces cognitive and neurobehavioral deficits (166, 167). While CCI enables reliably controlled injury across a range of severities, this model injury commonly induces post-traumatic apnea and cavitating tissue necrosis in and around the impact depression zone. Most common implementations of CCI require anesthesia.

Marmarou weight drop model (168, 169) and variant models utilize a mass that is dropped from a known height onto a metal plate attached to the skull of the animal subject. The head is placed on a deformable foam bed that allows the head to accelerate during the impact. The metal plate prevents skull fracture. This model reliably produces DAI, widespread axonopathy, TMI, as well as neuromotor and cognitive deficits (168–171). Mortality in this model results from protracted apnea and cerebral hypoxia secondary to brainstem trauma (171). The Marmarou weight drop model is conducted under anesthesia.

Closed-head impact model of engineered rotational acceleration model (CHIMERA) is a new variant of the extracranial CCI model (151). A pneumatically driven piston is used as an impactor to drive traumatic flexion of the cervical spine and rotational acceleration of the head. The resulting injury combines components of cervical whiplash and acceleration head injury. This model produces DAI, reactive microgliosis, release of inflammatory cytokines (TNFalpha, IL-1 beta), and hyperphosphorylated tau proteinopathy (151). The model injury also induces neuromotor and behavioral deficits and presumably results in cranial deformation and possibly cervical spine injury. As presently implemented, this injury model produces protracted periods of inactivity, motor dysfunction, and post-traumatic apnea in some animals. These responses are consistent with brainstem injury, spinal shock, and/or secondary hypotension (151, 172). The CHIMERA model requires anesthesia.

#### Animal Models of CTE

Exposure to explosive blast is known to induce TBI and CTE brain pathology in humans (103, 112, 134, 173). Goldstein et al. developed a mouse model of blast-related neurotrauma that reliably induces CTE-linked phosphorylated tau proteinopathy, neuroinflammation, diffuse astrocytosis, TMI, myelinated axonopathy, neurophysiological abnormalities, and learning-memory deficits that strikingly recapitulate acute and chronic effects of blast-related TBI and CTE in humans (112, 120). In this animal model of CTE, animal subjects are positioned orthogonal to the direction of the blast front with the thorax protected and the head exposed and free to rotate. Blast exposure in this and variant blast and impact models triggers abrupt onset of progressive phosphorylated tau proteinopathy that appears to spread throughout the brain over time (120, 129, 174–177). Restriction of head movement during blast exposure prevented neurobehavioral learning and memory deficits post-injury. This model was important in establishing the contribution of accelerative forces on the head as a driver of CTE pathology (112, 120). The model is compatible with 100% survivability and does not induce gross neuromotor impairment, post-exposure apnea, cervical spine trauma, or “blast lung” (112). While early implementation of the blast TBI-CTE model utilized anesthesia during blast exposure, recent studies are being conducted in non-anesthetized mice and demonstrate the same post-injury brain pathology and functional sequelae.

## Summary

Animal models of concussion, TBI, CTE, and other neurotrauma-related disorders are widely available and routinely deployed in research laboratories around the world. Effective and informative utilization of a given animal model requires consideration and implementation of four basic principles. First, the model must be grounded by clear definitions of the disease, condition, or endophenotype being modeled (i.e., nosology). This first principle is best served by focusing on specific clinical features that define a particular phenotype of the disease or condition under investigation. Second, the purpose and frame of reference of the animal model must be clearly understood and articulated (i.e., utility). Technical artifacts with potential to interfere with or modulate primary endpoints must be considered, routed out, or rigorously control. Model artifacts such as systemic anesthetic exposure are problematic for experimental studies that use laboratory animals to investigate concussion, TBI, or CTE. Third, concordance between key features of the animal model and the human disease or condition being modeled must be demonstrated (i.e., biofidelity). Fourth, experimental results observed in the animal model must be confirmed by correlative clinical findings in human subjects (i.e., validity). These principles are often given scant attention or neglected altogether. Adherence to these principles serves as a key bulwark against flawed interpretation of results, replication failure, scientific miscommunication, and confusion in the field. We anticipated that the implementation of these principles will accelerate translation of basic science discovery into clinical benefits for people affected by acute and chronic effects of neurotrauma.

## Author Contributions

MW drafted the manuscript, organized sections, and reviewed data from references to be included in this review. AF and OM reviewed models discussed in this review and made critical revisions of the manuscript. LG developed the concept of this review and wrote the manuscript. All coauthors reviewed and approved the paper and its contents.

## Conflict of Interest Statement

The authors declare that the research was conducted in the absence of any commercial or financial relationships that could be construed as a potential conflict of interest.

## Footnotes

^1^According to the CDC definition, these clinical signs can be used to support the diagnosis of mild TBI, but cannot be used to make the diagnosis in the absence of loss of consciousness or altered consciousness.
